# Chronic hepatitis E virus infection after living donor liver transplantation via blood transfusion: a case report

**DOI:** 10.1186/s40792-016-0159-0

**Published:** 2016-04-08

**Authors:** Takeshi Kurihara, Tomoharu Yoshizumi, Shinji Itoh, Norifumi Harimoto, Noboru Harada, Toru Ikegami, Yuki Inagaki, Yukio Oshiro, Nobuhiro Ohkohchi, Hiroaki Okamoto, Yoshihiko Maehara

**Affiliations:** Department of Surgery and Science, Graduate School of Medical Sciences, Kyushu University, 3-1-1 Maidashi, Higashi-ku, Fukuoka, 812-8582 Japan; Division of Gastroenterological and Hepatobiliary Surgery, and Organ Transplantation, Department of Surgery, Faculty of Medicine, University of Tsukuba, Ibaragi, Japan; Division of Virology, Department of Infection and Immunity, Jichi Medical University School of Medicine, Tochigi, Japan

**Keywords:** HEV, LDLT, Immunosuppression, Ribavirin

## Abstract

Although it occurs worldwide, hepatitis E virus (HEV) infection in developed countries is generally foodborne. HEV infection is subclinical in most individuals. Although fulminant liver failure may occur, progression to chronic hepatitis is rare. This study describes a 41-year-old man with liver cirrhosis caused by non-alcoholic steatohepatitis and hepatocellular carcinoma within the Milan criteria. His liver function was classified as Child-Pugh grade C. Living donor liver transplantation (LDLT) was performed, and he was discharged from the hospital on postoperative day (POD) 22. However, his alanine aminotransferase concentration began to increase on POD 60 and HEV infection was detected on POD 81. Retrospective assessments of stored blood samples showed that this patient became positive for HEV RNA on POD 3. The liver donor was negative for anti-HEV antibodies and HEV RNA. However, the platelet concentrate transfused into the liver recipient the day after LDLT was positive for HEV RNA. The patient remained positive for HEV infection for 10 months. Treatment with 800 mg/day ribavirin for 20 weeks reduced HEV RNA to an undetectable level. In conclusion, this report describes a patient infected with HEV through a blood transfusion after LDLT, who progressed to chronic hepatitis probably due to his immunosuppressed state and was treated well with ribavirin therapy.

## Background

Hepatitis E virus (HEV) is a member of the genus *Orthohepevirus* within the family *Hepeviridae* [1]. Approximately 20 million persons per year worldwide are estimated to be infected with HEV. Four major HEV genotypes can infect humans. HEV genotypes 1 and 2 are only found in humans, whereas HEV genotypes 3 and 4 are found in swine and other animals, infecting humans in developed countries through consumption of food [2].

Hepatitis E was previously regarded as a cause of acute hepatitis, with no progression to chronic hepatitis. More recently, however, progression to chronic hepatitis has been observed in immunocompromised hosts, including patients receiving solid organ transplants [3], those positive for human immunodeficiency virus (HIV) [4] and patients receiving chemotherapy for hematological malignancies [5].

Our group recently published the first nationwide survey about HEV infection among Japanese liver transplant recipients [6]. Two liver transplant recipients were diagnosed with HEV infection after living donor liver transplantation via blood transfusion. Here, we describe the clinical course of the patient who remained positive for HEV and was treated well with ribavirin therapy.

## Case presentation

A 41-year-old man with a history of liver cirrhosis caused by non-alcoholic steatohepatitis (NASH) and hepatocellular carcinoma (HCC) within the Milan criteria was referred to our hospital for possible living donor liver transplantation (LDLT) from his healthy 35-year-old brother. The patient was obese from childhood, with a peak body weight of 140 kg. He had been treated for HCC by radiofrequency ablation 2 years earlier and by transarterial chemoembolization 5 months earlier. Laboratory data included white blood cell count 3320/mL, hemoglobin 12.6 g/dL, platelet count 5.6 × 10^4^/mL, total protein 6.4 mg/dL, albumin 2.7 g/dL, creatinine 0.87 mg/dL, total bilirubin 3.7 mg/dL, aspartate aminotransferase (AST) 91 U/L, alanine aminotransferase (ALT) 53 U/L, sodium 137 mEq/L, ammonia, 135 mg/dL, and prothrombin time 32 %. The patient was classified as Child-Pugh grade C with 11 points, and his model for end-stage liver disease score was 20. He was negative for hepatitis B surface antigen and antibody to hepatitis C virus. Tumor markers included α-fetoprotein 5.1 ng/mL and PIVKA-II 10,848 U/mL.

Laparotomy revealed a cirrhotic liver and an enlarged spleen. The recipient’s liver was removed, and the donated right lobe graft was implanted. The graft weight was 798 g, corresponding to 57.4 % of the recipient’s standard liver weight. Splenectomy was also performed. The total operating time was 819 min, and total blood loss was 3450 mL. There are three hepatocellular carcinomas in the resected specimen. The largest tumor size was 20 mm in diameter. Histological examination showed a well-differentiated hepatocellular carcinoma growing in trabecular and focally pseudo-glandular patterns. The surrounding liver tissues showed micronodular cirrhotic change with mild chronic inflammatory infiltrate the fibrous stroma (A1F4) with 5 % macrovesicular steatosis, which was compatible with burn-out NASH.

On the day of surgery, the patient received transfusions of 20 units of red blood cell concentrates, 12 units of fresh frozen plasma, and 20 units of platelet concentrate. On postoperative day (POD) 1, he was transfused with 16 units of fresh frozen plasma and 20 units of platelet concentrate. Subsequent transfusions included four units of fresh frozen plasma on POD 2, two units of red blood cell concentrate on POD 9, and four units of red blood cell concentrate on POD 13 (Table 1).

**Table 1:** Details of blood transfusions

Date	Content	Units	Lot number
Day of LDLT	RCC	2	5333258575
	RCC	2	5642253634
	RCC	2	5646255421
	RCC	2	5642253633
	RCC	2	5155253493
	FFP	4	5033365354
	FFP	4	5033365353
	PC	10	5621364018
	RCC	2	5321250827
	RCC	2	5033264997
	RCC	2	5510248680
	RCC	2	5020264754
	RCC	2	5642253614
	FFP	4	5155347562
	FFP	4	5033365452
	PC	10	5020364469
POD 1	FFP	4	6204356192
	PC	10	5033368990
	FFP	4	5580359072
	PC	10	5020364476
	FFP	4	5580359150
	FFP	4	5222350235
POD 2	FFP	4	5101362480
POD 9	RCC	2	5006268035
POD 13	RCC	2	5112254264
	RCC	2	5112254256

*LDLT* living donor liver transplantation, *RCC* red cell concentrate, *FFP* fresh frozen plasma, *PC* platelet concentrate, *POD* postoperative days

**Fig. 1:**
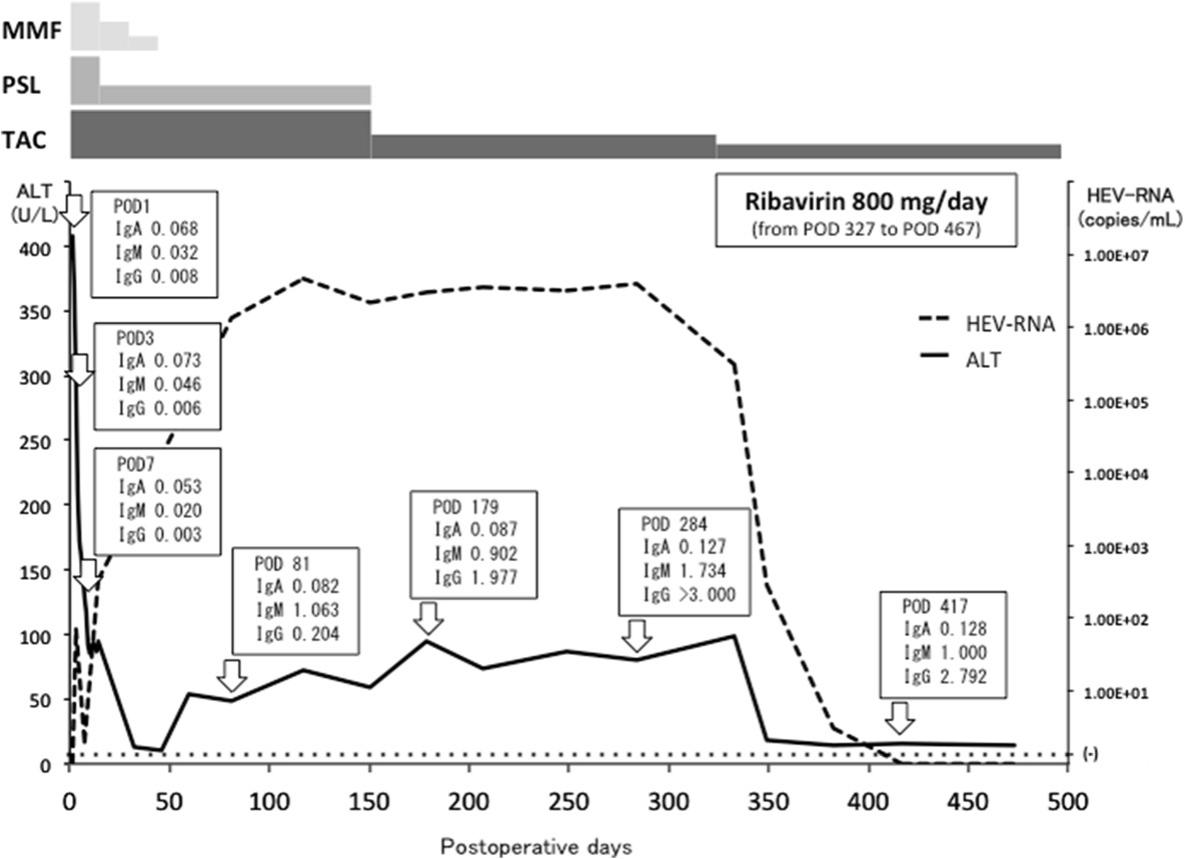
Postoperative ALT (U/L) transition; anti-HEV IgA, anti-HEV IgM, and anti-HEV IgG concentrations (OD at 450 nm) and HEV-RNA titers (copies/mL) in this patient. The patient became positive for HEV RNA on POD 3. Mildly abnormal ALT was observed on POD 60, with HEV RNA confirmed as being persistently positive. Ribavirin therapy was started on POD 327. ALT normalized on POD 382 and HEV RNA became undetectable on POD 417. The therapy was done until POD 467. HEV RNA remained negative on POD 536. *IgA* anti-HEV IgA, *IgM* anti-HEV IgM, *IgG* anti-HEV IgG, *MMF* mycophenolate mofetil, *PSL* prednisolone, *TAC* tacrolimus

Following LDLT, immunosuppression was started, including tacrolimus, mycophenolate mofetil, and steroids. The patient was discharged from the hospital on POD 22. Treatment with mycophenolate mofetil was discontinued on POD 32. His liver-enzyme became elevated (AST 46 U/L, ALT 56 U/L, total bilirubin 0.5 mg/dL) on POD 60. Immunosuppression was maintained including tacrolimus to the trough of 10 to 15 ng/mL and prednisolone 5 mg/day. At the same time, nationwide survey to clarify the presence of HEV infection after liver transplant in Japan was started and his blood samples taken on POD 81 showed positive for HEV RNA and IgG and IgM antibodies to HEV. To determine the time of HEV infection, stored blood samples taken just before transplantation and on PODs 1, 3, 7, and 14 were assayed for the presence of HEV RNA and anti-HEV antibodies. Blood samples from the donor were also assayed to determine whether infection was graft mediated. These assays showed that the patient first became positive for HEV RNA on POD 3, remaining positive thereafter. The donor sample was negative for anti-HEV antibodies and HEV RNA (Fig. 1).

### Analysis of the route of infection

The transplantation protocol of our institution does not permit patients to ingest raw meat or raw fish until 1 year after LDLT. This patient had not ingested such foods after LDLT, precluding the likelihood of foodborne infection with HEV.

Eliminating the possibilities of donor and foodborne sources of HEV suggested that this patient may have become infected through a blood transfusion. Assays of the transfused samples, supplied by Fukuoka Prefecture Red Cross Blood Center, showed that HEV RNA was present in the platelet concentrate transfused into this patient on POD 1 (lot number 5020364476).

### Clinical significance of the HEV genotype

The genomic sequences of the HEV strain detected in the patient and the transfused platelet concentrate were compared. Sequences compared included a 326-nucleotide (nt) 5′-terminal region of the open reading frame (ORF)1, a 505-nt region of the proline-rich hinge (V) domain within the ORF1, and a 412-nt region of the ORF2. The two strains differed by one nt in the ORF1 and by two nts in the V domain, but their ORF2 sequences were identical.

**Fig. 2:**
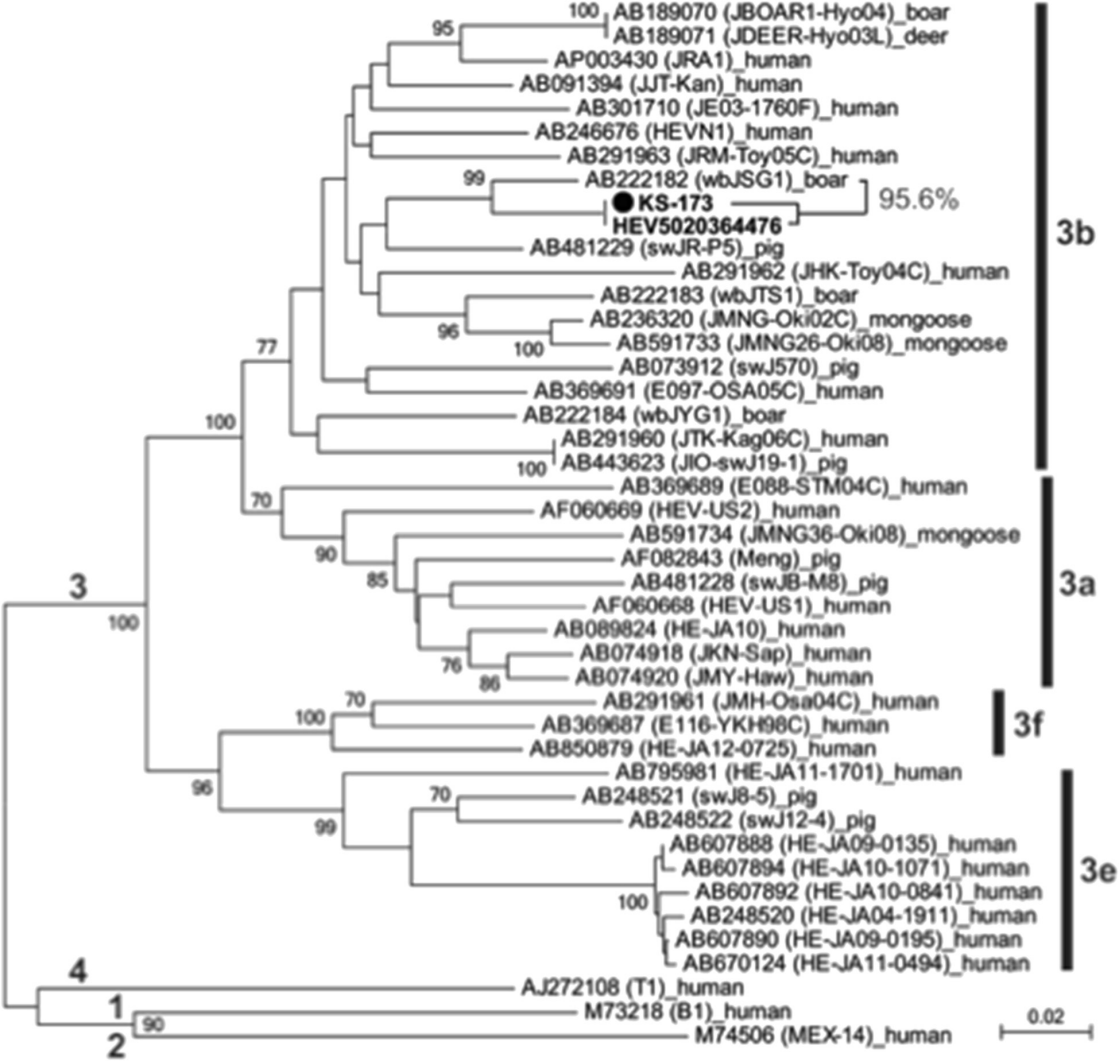
Phylogenetic tree of HEV constructed based on the 412-nt ORF2 sequence of 44 HEV strains, showing that the KS-173 strain in this patient was identical to the HEV strain (HEV5020364476) recovered from one of the transfused blood samples and shared the highest identity of 95.6 % with an HEV strain (wbJSG1) isolated from a wild boar that had been captured in Saga Prefecture, Kyushu Island, Japan

Phylogenetic analysis of the HEV strains, conducted according to the previously described method [7], revealed that the HEV strains in the transfused sample, and the patient were both classifiable into genotype 3 (subgenotype 3b) (Fig. 2). These two isolates were closest to an HEV strain (95.6 % identity) isolated from a wild boar that had been captured in Saga Prefecture on Kyushu Island of Japan. These findings suggested that the source of infection was HEV strains circulating on Kyushu Island, Japan.

### Antiviral treatment

Treatment with steroids was decreased gradually and discontinued on POD 150. Thereafter, immunosuppressive therapy was done with tacrolimus monotherapy and the dosage was reduced to the target trough of 5–10 ng/mL. The patient remained positive for both HEV RNA and anti-HEV antibodies on POD 284, more than 6 months after diagnosis of HEV infection. Although a liver biopsy was not obtained, this patient was considered a chronic carrier of HEV. Treatment with ribavirin 800 mg/day was started on POD 327. AST and ALT became normal on POD 382, and he became negative for HEV RNA on POD 417. The therapy was done until POD 467. HEV RNA remained negative on POD 536.

### Discussion

Chronic hepatitis E is characterized by mild derangement in liver enzymes, persistently detectable HEV RNA in serum and stool for >3 months, delayed/absent HEV seroconversion, and histological features of chronic viral hepatitis, such as dense lymphocytic periportal infiltrates with piecemeal necrosis [8]. Screening for HEV RNA in the sera of liver transplant recipients showed that the prevalence of HEV infection in Europe was <1 % [9, 10].

The inability to clear HEV viremia is directly related to the degree of immunosuppression of these patients at the time of infection. Therefore, infections that occur shortly after transplantation and rejection episodes, and when patients have low total lymphocyte counts, are more likely to become chronic. Treatment with tacrolimus, as opposed to cyclosporine, was also shown to be a risk factor for chronic HEV in posttransplant patients [11]. Our immunosuppression strategy was initiated using a protocol based on either tacrolimus (Prograf; Astellas Pharma, Tokyo, Japan) or cyclosporine A (Neoral; Novartis Pharma K.K., Tokyo, Japan) with steroids with or without mycophenolate mofetil. The target tacrolimus trough concentration was set at 10 ng/mL for 3 months after LDLT and 5–10 ng/mL thereafter. The target trough concentration of cyclosporine A was set at 250 ng/mL for 3 months after LDLT and 150–200 ng/mL thereafter. Methylprednisolone was initiated on the day of LDLT, after which the dosage was tapered and prednisolone substituted for 7 days after LDLT. Prednisolone treatment was tapered and discontinued 6 months after LDLT. Mycophenolate mofetil was beginning with 1–2 g/day on the day after LDLT; the dosage was tapered and discontinued 6 months after LDLT. In this case, immunosuppression including tacrolimus might have influenced the chronicity of HEV infection.

Chronic HEV infection is frequently related to the consumption of undercooked pork or, in some cases, mussels or game meat [12]. Transmission of HEV via an infected liver graft has also been reported [13]. Transfused blood products are another potentially important mode of acquisition. In the UK, it was recently estimated that one in 2848 blood donors has hepatitis E viremia at the time of donation [14]. The first case of HEV infection via a blood transfusion in Japan was reported in 2004 [15]. A nationwide survey of the prevalence of HEV infection in Japan showed that 431 (3.4 %) of 12,600 donated blood samples were positive for anti-HEV IgG [16]. Although all donated blood samples are not checked for HEV, samples donated on Hokkaido Island are assayed by PCR amplification of HEV specific sequences, as the prevalence of acute hepatitis E is higher in Hokkaido than elsewhere in Japan [17]. In the present, screening of donated blood for HEV has only been conducted in the Hokkaido area. Patients receiving transfusion include many immunocompromised patients, so screening of blood donors for HEV in other areas in Japan is probably desirable.

Immunocompromised patients diagnosed with hepatitis E should be closely followed up, with regular monitoring of blood HEV RNA viral load, liver function tests, and liver stiffness. Delay in the management of persistent viremia should be minimized due to the potentially rapid onset of irreversible liver injury. Reduction in immunosuppression should be considered for patients with HEV viremia persisting for more than 3 months. In a previous study, reduction in immunosuppression, especially of calcineurin inhibitor, led to HEV clearance in one third of patients [11]. The remaining two third patients required antiviral therapy. If viremia still persists, treatment with ribavirin should be considered, as ribavirin monotherapy results in a sustained virological response rate of 78 % in patients with chronic hepatitis E [18].

## Conclusions

This study describes a patient who was infected with HEV after LDLT and progressed to chronic hepatitis. HEV was transmitted to this patient by a blood transfusion following LDLT. Treatment with ribavirin was successful.

## Ethics approval and consent to participate

This study was approved by the Ethics Committee of Kyushu University (IRB approval number 26–37).

## Consent for publication

Written informed consent was obtained from the patient for publication of this case report.

## Abbreviations

HCC: hepatocellular carcinoma
HEV: hepatitis E virus
LDLT: living donor liver transplantation
ORF: open reading frame
POD: postoperative day
